# Green Downscaling of Solvent Extractive Determination Employing Coconut Oil as Natural Solvent with Smartphone Colorimetric Detection: Demonstrating the Concept via Cu(II) Assay Using 1,5-Diphenylcarbazide

**DOI:** 10.3390/molecules27238622

**Published:** 2022-12-06

**Authors:** Kullapon Kesonkan, Chonnipa Yeerum, Kanokwan Kiwfo, Kate Grudpan, Monnapat Vongboot

**Affiliations:** 1 Department of Chemistry, Faculty of Science, King Mongkut’s University of Technology Thonburi, Bangkok 10140, Thailand; 2 Center of Excellence for Innovation in Analytical Science and Technology for Biodiversity-Based Economic and Society (I-ANALY-S-T_B.BES-CMU), Chiang Mai University, Chiang Mai 50200, Thailand; 3 Department of Chemistry, Faculty of Sciences, Chiang Mai University, Chiang Mai 50200, Thailand

**Keywords:** coconut oil, natural solvent, downscaling extraction, colorimetric solvent extraction determination, copper (II), diphenylcarbazide, diphenylcarbazone, green chemical analysis

## Abstract

Coconut oil as a natural solvent is proposed for green downscaling solvent extractive determination. Determination of Cu(II) using 1,5-Diphenylcarbazide (DPC) was selected as a model for the investigation. Cu(II)-DPC complexes in aqueous solution were transferred into coconut oil phase. The change of the color due to Cu(II)-DPC complexes in coconut oil was followed by using a smartphone and image processing. A single standard concept was used for a series of Cu(II) standard solutions. A downscaling procedure using a 2 mL vial provided a calibration: color intensity = −142 [Cu(II)] + 222, (R^2^ = 0.98), 10% RSD. Using a well plate, a calibration was: color intensity = 61 [Cu(II)] + 68 (R^2^ = 0.91), 15% RSD. Both were for the range of 0–1 ppm Cu(II). Application of the developed procedure to water samples was demonstrated. The developed procedures provided a new approach of green chemical analysis.

## 1. Introduction

Solvent extraction spectrophotometric determination has long been deployed for various analytes. Spectrophotometric determination of Cd(II) using dithizone was performed by extracting the complex into chloroform [1]. Using 1,5-diphenylcabazide (DPC), Cu(II) could be determined spectrophotometrically after extracting into benzene [2]. Fe(II) and Fe(III) could be extracted into 1-pentanol using anthranilic acid for spectrophotometric determination [3]. Complexing with 4-(2-pyridylazo)-resocinol, Pb(II) was spectrophotometrically determined in isobutyl methyl ketone [4]. Extraction of Zn(II) with ethylthioacetoactate into ethyl acetate allowed Zn(II) determination [5]. For Sb(III) and Sb(V), the determination was made by using mandelic acid and malachite green with extraction into chlorobenzene solvent [6]. Spectrophotometric solvent extraction determination of Ni(II) with di-2-pyridyl ketone benzoylhydrazone reagent and chloroform was reported [7].

Due to concerns about toxicity of the organic solvents used for the extraction, replacement of once popular solvents was gradually implemented. For example, benzene was at one point replaced by toluene, then by carbon tetrachloride or chloroform. And gradually use of such solvents for these purposes has been declining [8,9]. One of the green analytical chemistry approaches engages with research for greener alternatives for toxic organic solvents to minimize unfavorable impacts [10,11,12,13].

Recently, vegetable oils have been viewed favorably, in applications for food [14,15,16], health [17,18,19], and cosmetic [20,21,22] purposes, due to their properties in biodegradability, and excellent environmental aspects such as low ecotoxicity and low toxicity toward humans, apart from their global availability [23]. Some vegetable oils, namely, rapeseed oil, peanut oil, and coconut oil, were reported for sample preparation of high performance liquid chromatography (HPLC) determination of polycyclic aromatic hydrocarbons (PAHs) by using the oils to extract PAHs from polluted quagmires [24]. Coconut oil offers some characteristics suitable to an organic solvent for extracting some species from the aqueous to the organic phase, compared to the conventional solvents, such as its dielectricity (dielectric constants of 2.2 for coconut oil, compared with 2.3 and 2.4 for benzene and toluene, respectively, see Table S1). Coconut oil contains higher unsaturated fatty acids among vegetable oils, especially lauric acid (C12:0) [23], apart from other fatty acids.

Recently, use of smartphones with image processing has been incorporated in cost-effective colorimetric determination as a part of modern green chemical analysis [25,26].

In this work, considering the high local abundance of coconut oil not only in Southeast Asia, but also in various tropical areas, and although it has been applied for solvent extraction in a sample preparation step for HPLC analysis of PAHs as above mentioned [24], our attempts were to introduce coconut oil as a natural alternative solvent to enable a new greener approach for solvent extraction colorimetric determination. Determination of Cu(II) using DPC was selected as a model for demonstrating this approach. Investigation of downscaling procedures employing a smartphone colorimetric determination was made for solvent extractive determination employing natural coconut oil solvent. This approach would lead to greener chemical analysis that provides benefits to the environment.

## 2. Results and Discussion

### 2.1. Reinvestigation for Cu(II) Extraction toward the Reaction between Cu(II) and DPC

**Figure 1:**
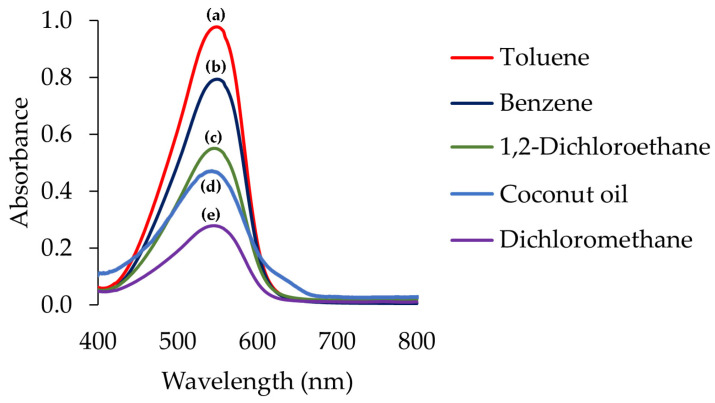
Visible spectra of Cu(II)-DPC complexes in solvents. Spectra: (a) toluene, (b) benzene, (c) 1,2-dichloroethane, (d) coconut oil, and (e) dichloromethane; for coconut oil, was normalized using OriginPro 2023 program (adjacent-averaging, points of window: 30).

Solvent extraction determination of Cu(II) employing DPC color reagent and benzene as solvent was reported [2]. Cu(II) reacts with DPC to form complexes, possibly with the oxidized form of DPC, diphenylcarbazone, in an alkaline aqueous medium [27,28]. The complexes would be then extracted into an organic phase. The method was selected as a model for this work. Following the conditions reported in [2] but with modifications, an investigation was made. Figure 1 illustrates the spectrum of the benzene extract. The absorption maximum exhibited at 549 nm, which agreed with the previous findings [2]. Other organic solvents, namely, toluene, DCM, and DCE were also investigated for extraction of Cu(II)-DPC complexes under the same concentrations and conditions. Some properties of the solvents are represented in Table S1. The absorption maxima of the extracts using the investigated solvents exhibited practically the same wavelength (545 ± 4 nm).

Coconut oil was investigated as an alternative solvent for this purpose. Using the same conditions as previously, the spectrum (in Figure 1) exhibited an absorption maximum at practically the same wavelength as with the above conventional organic solvents. With gas chromatographic information, it was found that all the chromatograms were identical (see Figure S1).

The absorbance (0.518) of Cu(II)-DPC extract in coconut oil was observed to be lower than that in toluene (0.977) and that in benzene (0.794) but it was in the same order as with in DCE (0.551) which was higher than with DCM (0.279).

Considering the toxicity of the solvents, coconut oil is considered to be nontoxic (hazard rating = 0), while the others are in the rating of level 2 apart from DCE being level 3. In the development of the solvent extraction determination techniques, benzene, toluene, and carbon tetrachloride were popularly used in the early stages. Coconut oil behaves differently from the other solvents in term of viscosity (see the values in Table S1). With this viscosity behavior, after the extraction, coconut oil may entail difficulty in transferring its aliquot for absorbance measurement by a conventional spectrophotometer. To overcome the difficulty, solvent extraction colorimetric determination of Cu(II) with DPC using coconut oil as solvent could make use of a smartphone camera, which is nowadays employed for colorimetry. Such a procedure with downscaling would also lead to green chemical analysis.

### 2.2. Downscaling Using a Small Vial

The design of downscaling the extraction was aimed to microliter scale operation. A small vial (2 mL capacity) that is usually used for chromatographic purposes, was used as an extraction container. The volumes of both aqueous and organic phases were designed for downscaling and were equally of 600 µL each. For solvent extraction, DCM has been used more favorably than toluene, benzene, and DCE; however, from the results in Table S1, DCE provided similar results to coconut oil. For this study, DCE and coconut oil were further investigated, and a single standard approach was incorporated. The coloring reagent (DPC) was added into the aqueous solution containing Cu(II) in buffer, instead of having DPC in organic phase as earlier. By doing this, Cu(II) should react with excess DPC to form Cu(II)-DPC complexes, which would be more efficiently extracted into the organic layer. The procedure was modified from earlier experiments. A standard Cu(II) solution (2.4 ppm) was used as a single standard solution for various Cu(II) standard concentrations in aqueous phase. For the 1 ppm Cu(II) final concentration of aqueous phase (600 µL), 250 µL of the standard Cu(II) solution (2.4 ppm) was pipetted into a small vial, followed by 150 µL DI water, then 100 µL Na_2_HPO_4_ buffer (1 M) and 100 µL DPC (10 mM), resulting in the final concentrations of 0.2 M and 1.67 mM for buffer and DPC, respectively. The mixture was mixed well before equilibrating with 600 µL DCE for 1 min by hand. A series of 0.2, 0.4, 0.6, 0.8, and 1 ppm Cu(II) final concentrations were prepared similarly by varying the volumes (50, 100, 150, 200, and 250 µL) of the single standard Cu(II) solution (2.4 ppm) and DI water volumes of 350, 300, 250, 200, and 150 µL, respectively. For blank, only 400 µL DI water without the standard Cu(II) solution was pipetted. After equilibrating, each vial was left to stand for phase separation. It was observed the clear phase septation could be observed nearly immediately after standing. Agitation was not observed in the experiments performed at the temperature of 35 ± 5 °C. The vials were photographed under the conditions described in Section 3.3 as illustrated in Figure S2a. After taking the photograph, each organic layer was transferred for absorbance measurement. The photograph was further processed for RGB mode [25], by ImageJ Software (version 13.0.6, National Institutes of Health, Bethesda, MD, USA) with RGB profile function. G intensity is observed to be the highest among Red (R), Green (G), and Blue (B) values. The calibration graphs can be obtained by a plot of G intensity versus Cu(II) concentrations while for conventional spectrophotometry, a calibration graph was from a plot of absorbance versus Cu(II) concentrations.

Similarly, coconut oil was used for the downscaling operation using a small vial for extraction under the same conditions as the procedure using DCE. Using coconut oil provided better sensitivity (considering from the slopes of calibrations), when using a smartphone detection, as shown in Table 1. Although coconut oil provided the better observed sensitivity, due to its viscosity, it is not practical to transfer the organic phase from the extraction vial into a cuvette for absorbance measurement using a spectrophotometer. The cumbersomeness was in addition to the fact that phase separation of the aqueous-coconut oil phases needed a longer period than that of aqueous-DCE phases. In Table 1, the results obtained by taking photographs are presented.

As detecting using a smartphone and image processing offers a conventional means for investigating, factors affecting the downscaling extraction determination of Cu(II) using DPC were further investigated.

The effect of DPC concentration was investigated by using the previous conditions for extraction of 1 ppm Cu(II) (final concentration of 600 µL), with varying final DPC concentrations in 600 µL aqueous solutions of 0.17, 0.83, 1.67, and 2.50 mM, providing mole ratios of DPC:Cu(II) of 11, 53, 106, and 160. It was observed that the G intensity of the extract became constant after the mole ratio (DPC:Cu(II)) of 53. The DPC concentration of 1.7 mM was used for further experiments. It was indicated that the ratio of DPC:Cu(II) was already in excess to extract Cu(II) into the DCE phase with maximum extraction efficiency. This was indicated by the constant G intensity.

Under the above conditions (with 1.7 mM DPC in 600 µL aqueous 1 ppm Cu(II) solution), apart from 1 min, shaking times of 0.5, 2, and 3 min were studied. It was observed that shaking times of 1 min or more resulted in the same G intensity. So, 1 min hand shaking time should be suitable for the extraction.

The volumes of DCE solvent were studied for 300 and 200 μL in addition to 600 µL for the extraction using the previous conditions. Using DCE of 600, 300, and 200 µL provided calibration equations (plot of G intensity versus Cu(II) concentration): G intensity = −134 [Cu(II)] + 234 (R^2^ = 0.97), G intensity = −236 [Cu(II)] + 219 (R^2^ = 0.99), and G intensity = −322 [Cu(II)] + 219 (R^2^ = 0.99), respectively. As expected, the lower volumes of DCE used would result in higher sensitivity (slopes). Although the sensitivity may be gained, however, there may be a problem in obtaining clear phase separation, as well as problems in transferring the organic phase for absorbance measurement using a conventional spectrophotometer.

It should be noted that using a smartphone camera for detection, 12 extraction vials can be handled for one run. It could be designed for 4 extraction vials for standards together with 4 samples in duplicate to be operated simultaneously for the extraction process. The 12 extraction vials after phase separation could be one-shot-photographed under the light-control-set up (see description in the Materials and Methods section). Figure S2 represents a one-shot-photograph of a set of 12 extraction vials. With this handling, a sample throughput would be gained compared to the conventional spectrophotometric procedure.

### 2.3. Downscaling Using a Well Plate

The downscaling extraction was further designed using a well plate aiming for lower volume (microliter scale operation) with higher sample throughput. In a 96-well plate, each well (capacity of 300 µL) would serve as an extraction container. An extraction was performed by having an equal volume of 120 µL each of aqueous and organic phases. Some preliminary investigations on light control for photography were made (see Section S3.2.1 in Supplementary Materials). For extraction, intensity (ΔG) of the extract could be then evaluated by ΔG intensity = G intensity_empty well_ − G intensity_extract well_.

In such a well, 120 µL aqueous solution containing Cu(II) standard (0.6 ppm) in Na_2_HPO_4_ buffer (0.2 M) and DPC (1.7 mM) was prepared by using an auto-pipet with a single standard solution approach similar to the above manipulation but with 5 folds lesser amounts. With using the auto-pipet, the solution would be mixed well. The aqueous solution was then equilibrated with 120 µL organic solvent. Using a multichannel auto-pipet, the equilibrating could be done for 8 extractions (8 wells) simultaneously. This would lead to enhanced sample throughput.

**Figure 2:**
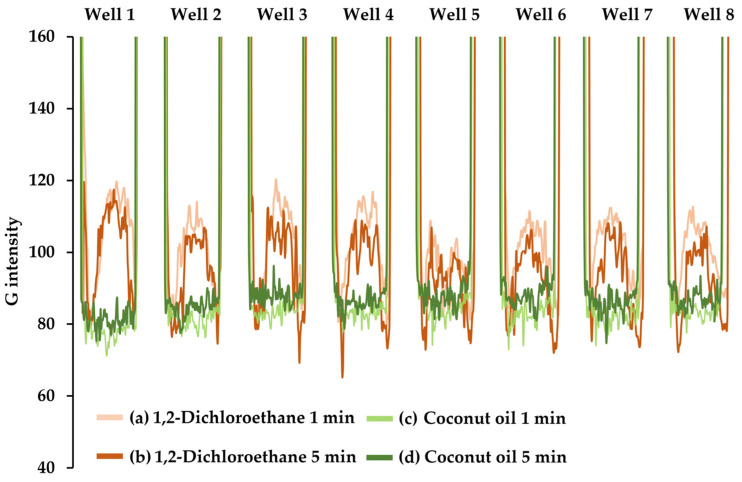
The intensity signal (G intensity) profiles of organic extract (from aqueous Cu(II)-DPC) using DCE at 1 min (a) and at 5 min (b) and coconut oil at 1 min (c) and at 5 min (d).

DCE as well as coconut oil were studied for extraction. As DCE has higher density than water, it would be expected that after phase separation, the DCE phase would be at the bottom of the well plate. Photographing of the experiment was made at the bottom. For coconut oil, it would be expected that the organic phase would be at the upper layer, due to its lower density. For this, the photograph was taken at the top of the well plate. Figure 2 represents the intensity signal (G intensity) profiles obtained using the above-described conditions. The profiles were at 1 and 5 min after the equilibrating. The profiles of both solvent extractions, in each well, exhibited two parts, namely at the middle and at the edge of the well, although it could be observed more on DCE than that of coconut oil. For coconut oil, a plateau shaped signal profile was pronounced more than the observation on the DCE’s. For coconut oil, a more reproducible profile pattern was observed. The profiles at 1 and 5 min were the same. Less reproducible profiles among the wells were seen for DCE and the signal profiles at 1 min showed a difference from 5 min. During the experiments, for DCE, it was observed that the DCE extract became gradually more viscous as a function of time. It could be that a component of the well plate (polystyrene) may dissolve (see Figure S4). The DCE was not used further.

Further study was made for standing time after the extraction. Under the above conditions, a standing period between 1–5 min was investigated. It was found that the same color intensity value of the organic (coconut oil) phase was obtained after 1 min. A standing time of 1 min was selected for further experiments.

For equilibrating aqueous-organic phases, this was done via using an auto-pipet by aspirating and dispensing from and to the well. The number of aspirating and dispensing cycles was studied. It was found that 15 or more cycles resulted in constant color intensity value of the organic extract. So, an operation with 15 cycles was selected for equilibrating the aqueous-organic phases.

Fixing the aqueous solution (Cu(II) and DPC in the buffer as above condition) of 120 µL, a series of the aqueous solutions containing 0, 0.2, 0.4, 0.6, and 0.8 ppm Cu(II) was extracted with 120 µL coconut oil. Experiments were repeated with the coconut oil of volumes of 60 and 40 µL. Three calibration graphs were obtained for the extractions with 120, 60, and 40 µL coconut oil. It was found that the calibration graph due to 40 µL coconut oil had the highest slope followed by the calibration graph of 60 µL coconut oil. The calibration graph with 120 µL coconut oil showed the lowest slope value but higher precision. This could be due to the coconut oil in the extraction playing a role in the organic phase adhering to the wells’ sidewalls and affecting the photography.

### 2.4. Analytical Performance

#### 2.4.1. Analytical Characteristics of the Downscaling Extraction Using Vial

The downscaling for extraction determination of Cu(II) using DPC involve handling solutions of less than 1 mL. Extraction performance and determination of Cu(II) were comparable to the conventional milliliter level extraction. A linear calibration (0–1 ppm Cu(II)) was absorbance = 0.90 [Cu(II)] + 0.12 (R^2^ = 0.99) when using a spectrophotometer, while the smartphone provided: G intensity = −133 [Cu(II)] + 173 (R^2^ = 0.99). The relative standard deviations (RSD) of both detections were less than 10% (0.4 ppm Cu(II)). Limits of detection (LODs) (3σ [29]) were estimated to be 0.02 and 0.1 ppm, while limits of quantitation (LOQs) (10σ [29]) being 0.1 and 0.4 ppm for spectrophotometric and smartphone detection, respectively. A set of 12 extraction vials could be handled in one run for extraction and detection by smartphone, see Figure S2. This made the procedure with smartphone detection more convenient than measuring absorbance by a spectrophotometer as no organic extract has to be transferred from the extraction vial to a cuvette. The 12 extraction vials of the set may comprise four standards for calibration and eight vials for four samples in duplicate, or various possibilities of other arrangements could be made. For the set of 12 extraction vials, a one run experiment may take 20 min, including building calibration and evaluation of the results.

#### 2.4.2. Analytical Characteristics of the Downscaling Extraction Using Well Plate

With a well plate, further downscaling could be operated with even smaller volumes (120 µL). The number of extraction units can be increased; 32 wells out of 96 wells would provide reasonably good results due to different light distribution occurring to some wells, see Figure S3. The 32 wells could be arranged for four standards in duplicate for duplicate calibration, in order to check the photographic conditions, and then 24 wells could hold 12 samples in duplicate (as illustrated in Figure S3b). Arrangement in other patterns may also be designed. After extraction, the plate was photographed in one shot. The operation of 32 extractions on one plate took 20 min, including image processing and evaluation. A calibration (0–1 ppm Cu(II)) was ΔG intensity = 61 [Cu(II)] + 68 (R^2^ = 0.91) with RSD of less than 15% (0.4 ppm Cu(II)). LOD (3σ) and LOQ (10σ) were evaluated to be 0.1 and 0.2 ppm, respectively. The operation using the well plate provided higher sample throughput than the throughput by the procedure using vials.

### 2.5. Applications

The proposed procedures have been applied to six local tap and drinking water samples. It was found that Cu(II) were less than LODs. All the samples were spiked with 0.4 ppm Cu(II). The results are presented in Table 2. The extraction procedure using a spectrometer was treated as a reference method. The results by all the procedures agreed each other and with the reference method. 

## 3. Materials and Methods

### 3.1. Chemicals and Reagents

All chemicals and reagents were analytical grade. Deionized (DI) water was used throughout. All glassware was soaked overnight in 10% *w*/*v* nitric acid solution (QRëC, Auckland, New Zealand), followed by rinsing with DI water prior to use.

A pure virgin coconut oil was obtained from Theppadungporn Coconut Co., Ltd., Bangkok, Thailand, and used without further treatment. The other chemicals included benzene (99.7%, Merck, Darmstadt, Germany), toluene (99.5%, VWR Chemicals, Solon, OH, USA), dichloromethane (DCM) (99.8%, Fisher Scientific, Waltham, MA, USA) and 1,2-dichloroethane (DCE) (99%, Loba Chemie, Mumbai, India).

As coconut oil posts freezing temperature of 25 °C [30], all experiments were carried out at a temperature of 35 ± 5 °C so that a clear organic solvent would be established.

A stock solution of 1000 ppm Cu(II) was prepared from copper sulfate (CuSO_4_·5H_2_O) (Kemaus, New South Wales, Australia). Working standard solutions of Cu(II) were prepared daily by appropriate dilutions of the stock solution with DI water. A solution of 10 mM DPC (Loba Chemie, Mumbai, India) was prepared by dissolving of 60 mg of the solid DPC in 12 mL of acetone (99.5%, Loba Chemie, Mumbai, India), followed by making up the volume with DI water in a 25 mL volumetric flask. A Na_2_HPO_4_ buffer solution (pH 9) was prepared by dissolving 8.90 g Na_2_HPO_4_·2H_2_O (Kemaus, New South Wales, Australia) in hot DI water; after complete dissolution, the volume was made up with DI water in a 50 mL volumetric flask.

### 3.2. Reinvestigation of Cu(II)-DPC Extracted into Organic Solvents

In a 60 mL polypropylene bottle, a mixture (5 mL) containing Cu(II) standard (0.6 ppm) and Na_2_HPO_4_ buffer (0.4 M) was equilibrated with DPC in benzene (0.4 mM, 7 mL). After vortexing for 1 min, it was left to stand for 10 min. An aliquot of benzene layer was transferred into a cuvette for recording visible absorption spectra, having a reagent blank as reference. The other organic solvents, namely, toluene, DCM, and DCE were also investigated for extraction of Cu(II)-DPC complexes followed the same concentration and conditions.

### 3.3. Downscaling Solvent Extraction Determination of Cu(II) Using DPC

#### 3.3.1. Using a Small Vial

Using an extraction vial (2 mL, clear color, KIMA, Bangkok, Thailand), 600 µL of aqueous solution containing Cu(II) with DPC in Na_2_HPO_4_ buffer was equilibrated with 600 µL of organic solvent. It was left to stand for phase separation before colorimetric measurement of the organic layer. The colorimetric measurements were made by using a smartphone camera (Galaxy Note 10 plus, Samsung, Seoul, Republic of Korea) and a spectrophotometer (Lambda35, PerkinElmer, Waltham, MA, USA).

**Figure 3:**
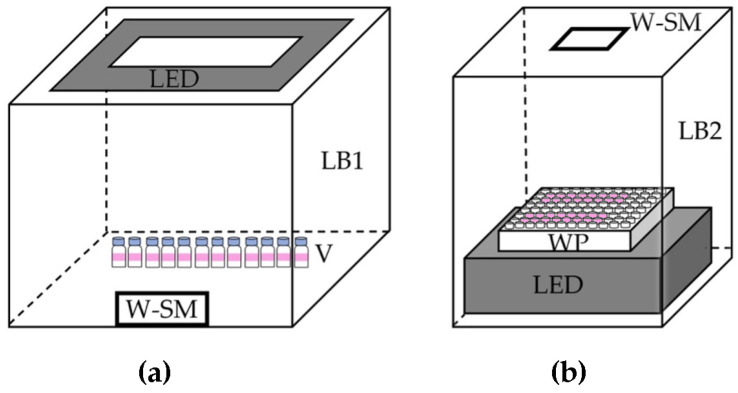
Illustration of the set ups for: (**a**) the extraction procedure using a small vial; (**b**) the extraction procedure using a well plate. LB1 = light control box (UDIOBOX UDIO BIZ, 40 × 40 × 40 cm, Bangkok, Thailand); V = small vial; LED = light emitting diode (LED) light source; W-SM = window for smartphone, LB2 = light control box (15 × 11 × 25 cm); LED = LED light source (LD-160, 14 × 5.7 × 9.5 cm, Lightdow, Shenzhen, China); WP = well plate; W-SM = window for smartphone. Note: the dimensions are not to scale.

For smartphone detection, the digital image of the extract vials was taken using smartphone camera with manual mode (ISO 200, Shutter Speed 3000, Zoom 1x and F1.5), under the light-controlled box see Figure 3a. The image was processed for the RGB profile using ImageJ Software (version 13.0.6, National Institutes of Health, Bethesda, MD, USA) with RGB profile function, by creating region of interest (ROI) with line across organic layer of every vial under investigated. Average G value was obtained from the ROI of each organic extract in the vial.

For spectrophotometric detection, after extraction, the 500 μL of DCE extract was transferred into a cuvette for absorbance (543 nm) measurement by a spectrophotometer.

#### 3.3.2. Using a Well Plate

Using a well plate (Nunc™ MicroWell™ 96-Well, Nunclon Delta-Treated, Flat-Bottom Microplate (167008), Thermo Scientific, Waltham, MA, USA), 120 µL of aqueous solution containing Cu(II) with DPC in Na_2_HPO_4_ buffer was equilibrated with 120 µL of organic solvent using multichannel auto-pipet (8 Channel pipettor (AP-8-200), Axygen, CA, USA). It was then photographed for colorimetric measurement of the organic layer. The colorimetric measurement was made by using a smartphone camera with manual mode as mentioned above, under the light-controlled box (see Figure 3b). The image was processed for the RGB profile using ImageJ Software (version 13.0.6, National Institutes of Health, Bethesda, MD, USA) with the function of RGB profile by having ROI with line across the well plate. Average G value could be obtained from the ROI of each organic extract in each well.

## 4. Conclusions

Coconut oil as a natural organic solvent is proposed as an alternative for solvent extraction colorimetric determination. Proof of the concept was demonstrated by the downscaling procedure for the determination of Cu(II) with DPC using a smartphone detector. Development of the downscaling procedures was based on using a small 2 mL vial and a 96 well microplate. Application to water samples was demonstrated. This new approach of green chemical analysis should be explored further for routine analysis in the real world. Various benefits would be obtained including cost-effective analysis, simple operation, on-site analysis, and environmentally friendly processes that are also less hazardous to the operator. Evaluating the developed procedures by the Green Analytical Procedure Index (GAPI) approach [31] (see Figure S5), extraction with coconut oil using well plates was the greenest, while the downscaling procedure using vials was greener compared to the traditional procedure [2]. It is very much of interest to investigate using other vegetable oils for green solvent extraction colorimetric determination, as a new approach for cost-effective green chemical analysis. This approach also supports the United Nations Sustainable Development Goals (SDGs).

## Figures and Tables

**Table 1:** Calibration graphs obtained from extraction of Cu(II) using DPC in a small vial.

Solvent	Calibration Equation	
	Conventional Spectrophotometry	Image Processing
1,2-Dichloroethane	Absorbance = 0.58 [Cu(II)] + 0.08, (R^2^ = 0.98)	G intensity = −134 [Cu(II)] + 234, (R^2^ = 0.97)
Coconut oil	−	G intensity = −142 [Cu(II)] + 222, (R^2^ = 0.98)

**Table 2:** Assay of Cu(II) in tap and drinking water samples spiked with 0.4 ppm Cu(II).

**Samples**	**Vial Procedure**				**Well Plate Procedure**	
	**Visible Spectrophotometer**		**Smartphone Camera**		**Smartphone Camera**	
	**Cu Found (ppm)**	**%Recovery**	**Cu Found (ppm)**	**%Recovery**	**Cu Found (ppm)**	**%Recovery**
1	0.41± 0.02	103 ± 3.70	0.41 ± 0.02	103 ± 4.88	0.34 ± 0.01	85 ± 1.49
2	0.34 ± 0.00	85 ± 0.00	0.44 ± 0.00	110 ± 0.00	0.40 ± 0.00	103 ± 1.05
3	0.40 ± 0.01	100 ± 1.27	0.45 ± 0.01	113 ± 1.12	0.40 ± 0.03	100 ± 7.41
4	0.37 ± 0.01	93 ± 1.37	0.40 ± 0.01	100 ± 2.50	0.45 ± 0.00	113 ± 0.97
5	0.37 ± 0.01	93 ± 1.37	0.43 ± 0.02	108 ± 3.53	0.40 ± 0.01	100 ± 3.23
6	0.41 ± 0.01	103 ± 2.44	0.44 ± 0.01	110 ± 1.15	0.34 ± 0.01	85 ± 3.18

## Data Availability

All the data are reported in this manuscript and supplementary materials.

## Supplementary Materials

The following supporting information can be downloaded at: https://www.mdpi.com/article/10.3390/molecules27238622/s1, Table S1: Some properties of the solvents and spectrophotometric information of Cu(II)-DPC complexes in the solvents; Figure S1: Chromatographic chromatograms of different coconut oils: (a) CHAOKOH brand lot1 (manufacture date February 2020), (b) CHAOKOH brand lot2 (manufacture date August 2020), (c) COCOLOVE brand, and (d) KING ISLAND brand; Figure S2: Extraction of Cu(II) via vial procedures using (a) DCE solvent and (b) coconut oil solvent; Figure S3: The well plate used for the extraction, (a) the empty well plate; (b) the well plate after extraction indicating with labels of standards and samples; Figure S4: DCE extract observed to be more viscous as function of time (extraction of aqueous Cu(II)-DPC into DCE in a polystyrene well plate; Figure S5: Evaluating the developed procedures by GAPI: (a) traditional [2]; (b) the developed procedure using vial with DCE; (c) the developed procedure using well plate with coconut oil [32,33,34,35,36,37,38,39,40,41,42].
