# Supplementary material for: Green Downscaling of Solvent Extractive Determination Employing Coconut Oil as Natural Solvent with Smartphone Colorimetric Detection: Demonstrating the Concept via Cu(II) Assay Using 1,5-Diphenylcarbazide
Source: Molecules. 2022 Dec 6;27(23):8622. doi: 10.3390/molecules27238622 (PMC9740733; doi:10.3390/molecules27238622)
Supplement: Supplementary file 1 [file molecules-27-08622-s001.zip › molecules-2049042-supplementary.pdf]

Supplementary Materials

# Green Downscaling of Solvent Extractive Determination Employing Coconut Oil as Natural Solvent with Smartphone Colorimetric Detection: Demonstrating The Concept Via Cu(II) Assay Using 1,5-Diphenylcarbazide

**Kullapon Kesonkan [1], Chonnipa Yeerum [1], Kanokwan Kiwfo [2] , Kate Grudpan [2,3]* and Monnapat Vongboot [1,*]**

1 Department of Chemistry, Faculty of Science, King Mongkut's University of Technology Thonburi, Bangkok 10140, Thailand
2 Center of Excellence for Innovation in Analytical Science and Technology for Biodiversity-Based Economic and Society (I-ANALY-S-T_B.BES-CMU) Chiang Mai University, Chiang Mai 50200, Thailand
3 Department of Chemistry, Faculty of Sciences, Chiang Mai University, Chiang Mai 50200, Thailand.
* Correspondence: kgrudpan@gmail.com (K.G.); sumalee.tan@kmutt.ac.th (M.V.)

## 1. Properties of the Solvents

**Table S1.** Some properties of the solvents and spectrophotometric information of Cu(II)-DPC complexes in the solvents.

| Solvent | Density (g mL$^{-1}$) | Viscosity (cP) | Dielectric Constant | Hazard Rating | The Maximum Wavelength (nm) | Absorbance |
|---|---|---|---|---|---|---|
| Benzene | 0.89 [a] | 0.56 [b] | 2.3 [a] | 2 [f] | 549 | 0.794 |
| Toluene | 0.87 [a] | 0.52 [b] | 2.4 [a] | 2 [g] | 549 | 0.977 |
| Dichloromethane | 1.34 [a] | 0.41 [c] | 9.1 [a] | 2 [h] | 545 | 0.279 |
| 1,2-Dichloroethane | 1.26 [a] | 0.72 [b] | 10.4 [a] | 3 [i] | 545 | 0.551 |
| Coconut oil | 0.91 [d] | 43.30 [d] | 2.2 [e] | 0 [j] | 543 | 0.518 |

[a, b, c, d, e, f, g, h, i] and [j] from references [32], [33], [34], [35], [36], [37], [38], [39], [40] and [41], respectively.

## 2. Characterization of Coconut Oil

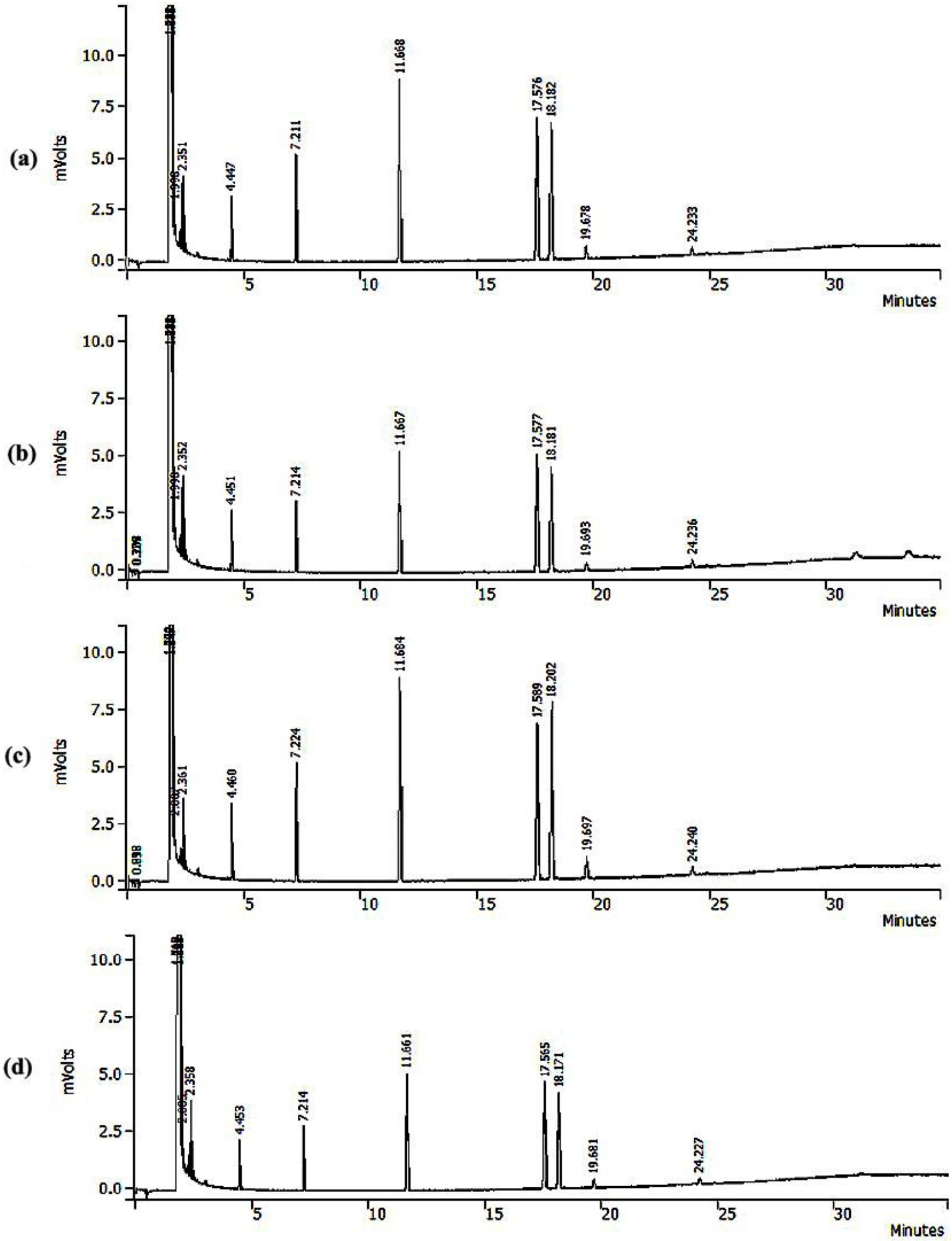


**Figure S1.** Chromatographic chromatograms of different coconut oils: (a) CHAOKOH brand lot1 (manufacture date February 2020), (b) CHAOKOH brand lot2 (manufacture date August 2020), (c) COCOLOVE brand, and (d) KING ISLAND brand.

Conditions

The coconut oil was characterized by gas chromatograph based on the base-catalyzed derivatization of fatty acid method [42]. Briefly, an exact amount of coconut oil (about 60 mg) was weighed into a small vial. Then, 1 mL of hexane, and 4 M potassium hydroxide in methanol were added and heated (50 °C) with magnetic stirring (100 rpm) for 30 min. The mixture was cooled to room temperature. DI water was vigorously added. An aliquot of the organic layer, after phase separation, was taken for the analysis using a gas chromatograph (Varian, CP-3800, USA) fitted with FID detector, with a polar capillary column CP-Wax52, injection temperature of 200 °C, the initial temperature of 150 °C with

temperature increasing rate 2 °C min$^{-1}$ until 210 °C. Chromatograms of different coconut oils are illustrated in Figure S1.

## 3. Downscaling Solvent Extraction of Cu(II) Employing DPC

### *3.1. Using a Small Vial*

**(a)**

**(b)**

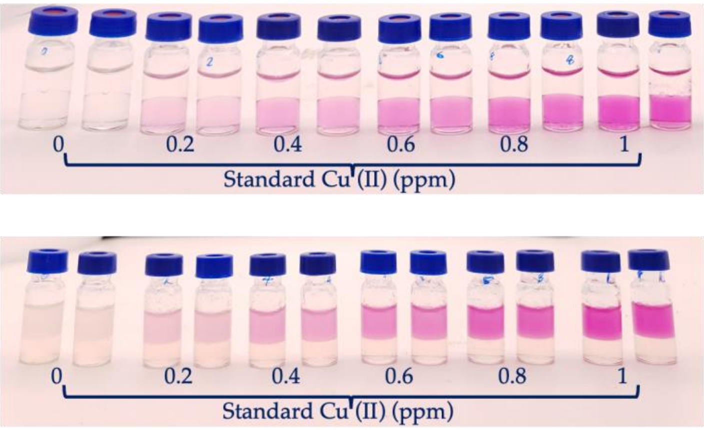


**Figure S2.** Extraction of Cu(II) via vial procedures using (a) DCE solvent and (b) coconut oil solvent.

### *3.2. Using a Well Plate*

#### 3.2.1. Investigation for Detection Condition by Using a Smartphone Camera

Investigation for detection condition by using a smartphone camera was made by placing 240 µL of a dye solution (rose pink, 1/500 of original concentration) into the 96 wells of the plate. The well plate was put in a light control box for photographing as set up illustrated in Figure 3b in the Materials and Methods section. An empty well plate was also photographed. The photographs were processed for G intensity. It was observed that not all of the 96 wells hosted a homogeneous brightness, as indicated in Figure S3a. For further experiments, only 32 wells would be deployed (as indicated in Figure S3b). The G intensity of each of 32 wells was laid in the mean G intensity ± standard deviation and the G intensity of the well would be evaluated by subtracting the G intensity of the empty well by the G intensity of the well with solution.

### 3.2.2. Design for Using the Well Positions in Downscaling Solvent Extraction

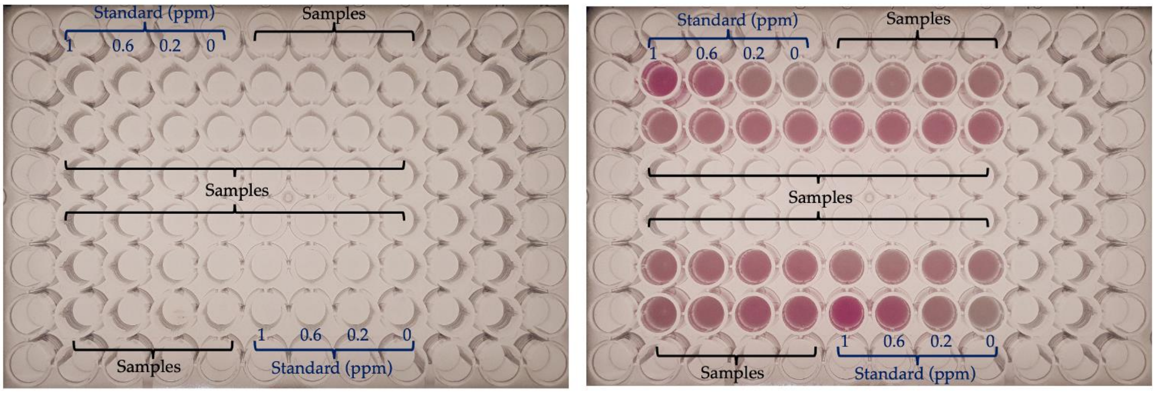


**(a)** **(b)**

**Figure S3.** The well plate used for the extraction, (a) the empty well plate; (b) the well plate after extraction indicating with labels of standards and samples.

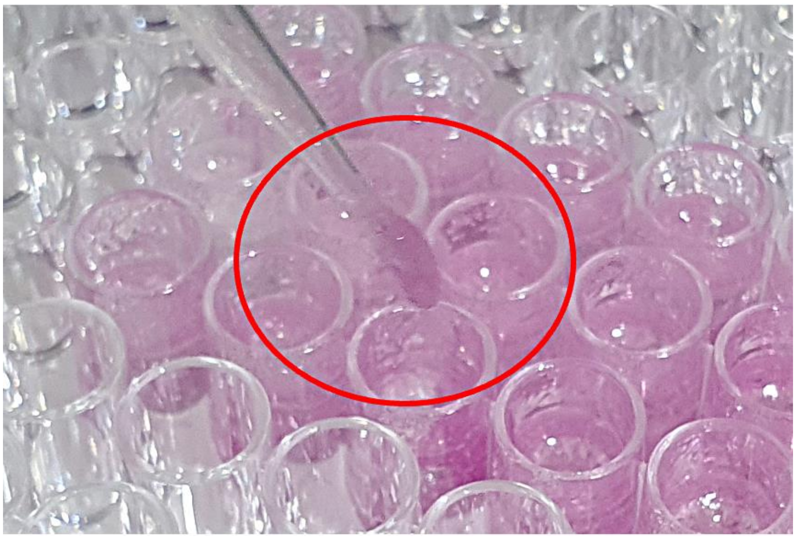

**Figure S4.** DCE extract observed to be more viscous as function of time (extraction of aqueous Cu(II)-DPC into DCE in a polystyrene well plate.

## 4. Evaluating the Developed Procedure by Green Analytical Procedure Index (GAPI) Approach

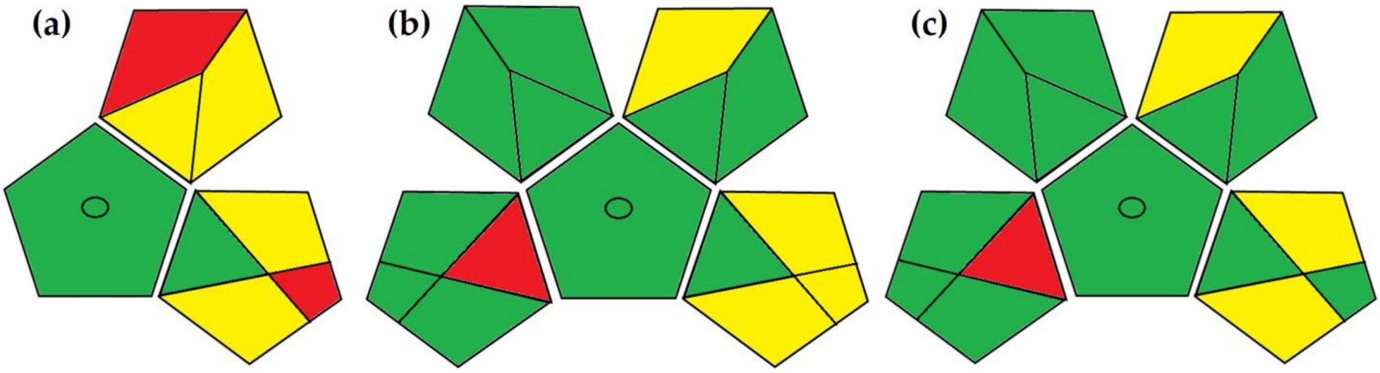


**Figure S5.** Evaluating the developed procedures by GAPI: (a) traditional [2]; (b) the developed procedure using vial with DCE; (c) the developed procedure using well plate with coconut oil.
